# Introduction to the Special Issue: The ecology and genetics of population differentiation in plants

**DOI:** 10.1093/aobpla/plab057

**Published:** 2021-09-06

**Authors:** F Xavier Picó, Mohamed Abdelaziz, Antonio R Castilla

**Affiliations:** 1 Departamento de Ecología Integrativa, Estación Biológica de Doñana (EBD), Consejo Superior de Investigaciones Científicas (CSIC), 41092 Sevilla, Spain; 2 Departamento de Genética, Universidad de Granada, 18071 Granada, Spain; 3 Department of Fisheries and Wildlife, College of Agriculture & Natural Resources, Michigan State University, East Lansing, MI 48824, USA; 4 Centre for Applied Ecology ‘Prof. Baeta Neves’, InBIO, School of Agriculture, University of Lisbon, 1349-017 Lisbon, Portugal

**Keywords:** Common garden experiments, epigenetics, local adaptation, molecular markers, next-generation sequencing, phenotypic plasticity, quantitative traits

## Abstract

Population differentiation is a pervasive process in nature. At present, evolutionary studies on plant population differentiation address key questions by undertaking joint ecological and genetic approaches and employing a combination of molecular and experimental means. In this special issue, we gathered a collection of papers dealing with various ecological and genetic aspects of population differentiation in plants. In particular, this special issue encompasses eight research articles and two reviews covering a wide array of worldwide environments, plant functional types, genetic and genomic approaches, and common garden experiments to quantify molecular and/or quantitative trait differentiation in plant populations. Overall, this special issue stresses the validity of traditional evolutionary studies focused on plant populations, whilst emphasizing the integration of classical biological disciplines and state-of-the-art molecular techniques into a unique toolkit for evolutionary plant research.

## Teaser

Several ecological and genetic processes acting in concert account for population differentiation, which is a pervasive process in nature that may eventually lead to speciation. In this special issue, we gathered a collection of papers dealing with various ecological and genetic aspects of population differentiation in plants covering a wide array of worldwide environments, plant functional types, genetic and genomic approaches, and common garden experiments to quantify molecular and/or quantitative trait differentiation in plant populations.

## Introduction

Mutation, anisotropic migration, random genetic drift and natural selection shape among- and within-population patterns of genetic diversity at different spatial and temporal scales, which steadily promote population differentiation, and in the end, may lead to speciation. This well-accepted statement identifies the major processes underlying evolutionary change, which is one of the major contributions of the Modern Synthesis that put together natural selection and Mendelian genetics under the umbrella of population genetics almost a century ago (Hancock *et al.* 2021). It also highlights the interest of evolutionary biologists in the relationship between genetic variation and the environment, which requires the combination of ecological fieldwork and laboratory genetics (Lowe *et al.* 2004). Such a combination of disciplines, first coined as ecological genetics by Ford (1964), has evolved towards ecological genomics (Ungerer *et al.* 2008) by incorporating technical advances in high-throughput sequencing, genotyping, genome-wide expression profiling and bioinformatics. Thus, evolutionary biologists currently have a wide array of resources to disentangle the genetic mechanisms underlying the response of organisms to the multiple biotic and abiotic challenges determining the genetic composition of populations and their differentiation.

The collection of papers in this special issue—‘The Ecology and Genetics of Population Differentiation in Plants’—provides insightful examples of the ecological genetics and ecological genomics of various plant species from different environments to tackle the causes and consequences of population differentiation. Each paper focuses on different aspects of population differentiation, from unravelling the evolutionary history to the design of conservation plans for the study species. Nevertheless, all the papers in this special issue share common ground. In particular, they undertake joint ecological and genetic/genomic approaches to address evolutionary questions and quantify the process of differentiation based on molecular markers and/or quantitative trait variation.

**Figure 1.**
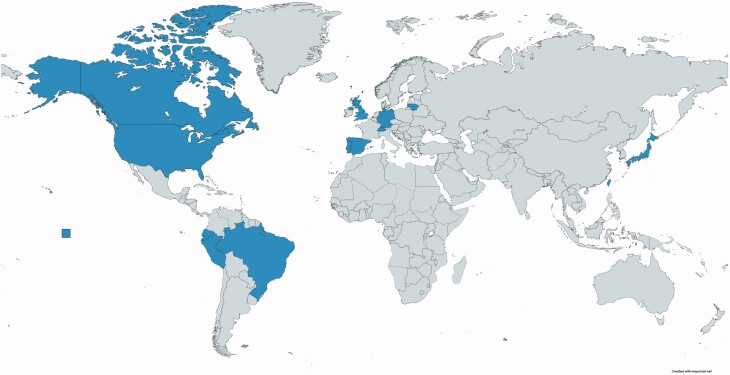
Map of contributing countries (authors’ affiliations and/or study regions) in America (Canada, USA, Peru, Ecuador and Brazil), Europe (Portugal, Spain, Switzerland, Germany, UK and Lithuania) and Asia (Japan and Taiwan).

**Figure 2.**
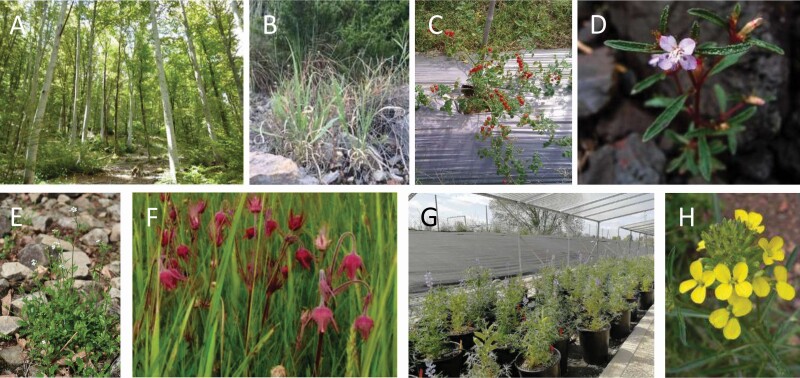
Pictures of some of the study species: (A) *Betula pendula*, (B) *Panicum hallii*, (C) *Solanum pimpinellifolium*, (D) *Brasilianthus carajensis*, (E) *Arabidopsis halleri*, (F) *Geum triflorum*, (G) *Lupinus angustifolius* and (H) *Erysimum mediohispanicum*. Pictures provided by the authors of each article of the special issue.

This special issue is the result of the session ‘Evolutionary Ecology in Terrestrial, Aquatic and Marine Environments’, held in the past SIBECOL meeting (4–7 February 2019, Barcelona, Spain) and organized by the guest editors with the sponsorship of *AoB PLANTS*. Up to 25 research groups, including 54 authors from 11 countries (Fig. 1), contributed to this special issue. Studies covered various world regions: coastal areas of Peru and Ecuador (Lin *et al.* 2020), mountains in Europe and Japan (Honjo and Kudoh 2019), the Iberian Peninsula (Matesanz *et al.* 2020; Medrano *et al.* 2020; Muñoz-Pajares *et al.* 2020), the Amazonian savannas (Silva *et al.* 2020), North America (Yoko *et al.* 2020; Palacio-Mejía *et al.* 2021) and European temperate forests (Solé-Medina *et al.* 2020). Furthermore, plant functional types were also diverse (Fig. 2), encompassing annual herbs (Lin *et al.* 2020; Matesanz *et al.* 2020; Silva *et al.* 2020), a biennial herb (Muñoz-Pajares *et al.* 2020), perennial herbs (Honjo and Kudoh 2019; Medrano *et al.* 2020; Yoko *et al.* 2020), a C_4_ grass (Palacio-Mejías *et al.* 2021) and a deciduous tree (Solé-Medina *et al.* 2020).

## Ecology and Genetics: Two Sides of the Same Evolutionary Coin

The duality of ecological and genetic/genomic approaches is the most prominent feature of all papers compiled in this special issue. For example, Lin *et al.* (2020) combined RAD (Restriction-site Associated DNA) sequencing and species distribution modelling to explore the niche and genetic differentiation of the wild tomato *Solanum pimpinellifolium* (Solanaceae) in South America. This allowed the authors to hypothesize about the origin of the species, which remained controversial. In addition, they identified the major ecological factors accounting for demographic behaviour and estimated the genetic offset, that is, whether the current genetic composition is maladaptive to future environments (Lin *et al.* 2020). Silva *et al.* (2020) also used RAD sequencing to identify neutral and adaptive genetic structure in the endemic herb *Brasilianthus carajensis* (Melastomataceae) from Amazonian Savannas. The authors not only characterized the population differentiation of a recently discovered species, but also employed genomic data to outline conservation/management actions to prevent the loss of unique genetic variation and maximize species resilience to future environmental change (Silva *et al.* 2020).

Furthermore, Muñoz-Pajares *et al.* (2020) combined nuclear microsatellites, demographic field data and a simulation model to explore the neglected concept of within-population temporal migration and its effect on genetic structure in the biennial plant *Erysimum mediohispanicum* (Brassicaceae) in SE Spain. Muñoz-Pajares *et al.* (2020) stressed the impact that within-population heterogeneity, such as among-individual variation in life cycle, may have on genetic structure mediated by temporal migrants. In the same geographic area, Medrano *et al.* (2020) compared genetic (DNA sequence variants) and epigenetic (DNA cytosine methylation variants) diversity in a set of phylogenetically closely related but ecologically disparate plant species. This study provided significant evidence on the important role that epigenetic diversity may have to account for adaptation, but also highlighted the complex role of epigenetic variation as a source of phenotypic diversity with evolutionary relevance (Medrano *et al.* 2020). Finally, Palacio-Mejías *et al.* (2021) evaluated large-scale geographic patterns of within-species genomic diversity in the C_4_ grass *Panicum hallii* (Poaceae) across its distribution in North America (Mexico and USA) also merging RAD sequencing and species distribution models. The authors identified genetic clusters that followed the main ecoregions of the species across its range, and pinpointed South Texas as a major hotspot of genetic diversity with the co-occurrence of all genetic clusters and admixture between the two *Panicum* varieties of study.

This special issue also covers the complementarity between ecology and genetics by using experimental means, which is an aspect of paramount importance in ecological genetics. For example, Matesanz *et al.* (2020) conducted an outdoor common garden experiment in Central Spain to evaluate the response of inbred maternal families of the legume *Lupinus angustifolius* (Fabaceae) to two ecologically meaningful water availability treatments. The common garden experiment tested the effect of drought treatments on plant traits and allowed the authors to identify cogradient variation in drought-related traits, that is, plasticity to drought was in most cases in the same direction as among-population quantitative genetic differentiation (Matesanz *et al.* 2020). Furthermore, Yoko *et al.* (2020) also undertook a common garden experiment in North Dakota (USA) to quantify genetic differences of traits attributed to broad regional- and population-level effects in *Geum triflorum* (Rosaceae). Yoko *et al.* (2020) applied their results for restoration-oriented purposes, in particular for considering the scale over which genetic differences may have evolved to establish seed transfer guidelines among populations. In addition, Solé-Medina *et al.* (2020) evaluated how variation in early fitness traits influenced the adaptive response of the tree *Betula pendula* (Betulaceae) in three common garden experiments across a substantial part of the species’ latitudinal range in Europe (Spain, Germany and Lithuania). In their study, Solé-Medina *et al.* (2020) quantified how important increasingly dry conditions may be to limit the regeneration capacity of the species, which has important implications for our understanding of global climate change on plants at wide geographical scales. Finally, Palacio-Mejías *et al.* (2021) quantified phenotypic divergence between two *P. hallii* varieties (var. *hallii* and var. *filipes*) in a common garden experiment in South Texas (USA). In this case, the authors used an experimental approach as a complement to characterize the divergence between the two *Panicum* varieties of study, finding important differences between them in morphological features, cold stress tolerance and lifespan (Palacio-Mejías *et al.* 2021).

The two reviews of this special issue focused on topics enabled by rapid technological developments. In the first review, Honjo and Kudoh (2019) proposed the use of transcriptome analyses, based on RNA-Seq methods, to treat quantitative measures of gene expression as conventional measures of phenotypic traits in the perennial *Arabidopsis halleri* (Brassicaceae). On top of architectural and life-history data, gene expression data can also be used to assess genetic differentiation and local adaptation for samples from field populations, common garden experiments and/or reciprocal transplant experiments (Gould *et al.* 2018; Honjo and Kudoh 2019). This review also stressed the utmost importance of choosing the right material for ecological genetic/genomic investigations, as already pointed out since the birth of the discipline (Lowe *et al.* 2004). In the other review, Denney *et al.* (2020) highlighted an understudied aspect of population differentiation in plants: micro-environmental variation as a driver of within-population variation in trait expression and genetic/genomic diversity in natural plant populations, including its buffering effects for the negative impact of rapid anthropogenic climate change on plant population viability. The combination of new techniques in remote sensing, infrared imaging, data loggers and computer algorithms designed to process the information allows the characterization of micro-environmental variation over multiple spatial and temporal scales (Denney *et al.* 2020).

## The Concept of Population: Easy to Understand, Hard to Tackle

The primary subject matter of this special issue—population differentiation—exemplifies the confluence of biological disciplines into the concept of population. Although there exist three major paradigms (Waples and Gaggiotti 2006) encompassing all accepted definitions of a population—the ecological (emphasizing demographic cohesion), the evolutionary (emphasizing reproductive cohesion) and the statistical paradigm (emphasizing sampling cohesion)—in practice, researchers design their sampling schemes in a way relevant to their goals beyond the concept of population. For example, one effective way to capture individual-level genetic diversity across wide geographical areas is to work with accessions. This approach has proven to be successful in major plant model systems, such as *Arabidopsis thaliana* (The 1001 Genomes Consortium 2016), rice (Wang *et al.* 2018), corn (Liu *et al.* 2020) and wheat (Sansaloni *et al.* 2020) to name a few. In this special issue, the accession-based approach was used in three papers and very adequate to address regional-scale differentiation patterns. For example, Lin *et al.* (2020) adopted an accession-based approach including 94 accessions of wild tomato sampled across the wide environmental diversity across South America. Silva *et al.* (2020) sampled up to 150 individuals of their endemic study herb from all over its known distribution range in Amazonian Savannas. Finally, Palacio-Mejia *et al.* (2021) undertook a mixed sampling strategy including 480 individuals of their study grass from 76 locations (1–22 individuals per location) across the natural distribution of the two subspecies of interest in North America. These three studies, given their specific goals, prioritized the analysis of the genetic diversity across their species’ distribution over the amount and distribution of genetic diversity within and among populations.

In addition to population differentiation, the rest of the studies considered patterns of within-population variation as well. These studies required the quantification of the within-population level of variation, which forced researchers to select a smaller number of populations and increase the number of individuals per population. For example, Matesanz *et al.* (2020) selected four populations (21 maternal plants per population) of their annual legume from climatically distinct regions to disentangle the genetic and environmental components of trait variation by common garden experimental means including drought treatments. Medrano *et al.* (2020) chose three populations (23–40 individuals per population) as replicates of seven pairs of congeneric species, each pair consisting of one endemic, restricted-range species associated with stressful environments, and one widespread species occupying more favourable habitats to compare genetic and epigenetic patterns of variation. Muñoz-Pajares *et al.* (2020) collected material of their biennial plant from 30–40 vegetative and reproductive individuals from four populations over 3 years to quantify the effect of temporal migrants on genetic differentiation patterns. Yoko *et al.* (2020) sampled 22 populations (40 maternal seed families per population) of their perennial plant from distinct ecoregions in North America to differentiate regional and local patterns of trait variation by experimental means. Finally, Solé-Medina *et al.* (2020) sampled 12 populations of silver birch (11–25 trees per population) over 2 years to explore variation in early fitness traits in a large-scale common garden experiment across Europe.

## Differentiation: A Driving Force for Adaptation and Speciation

We can define differentiation as the accumulation of trait values—related to the phenotype, the genotype and/or to the ecological characterization of organisms, such as patterns of niche occupancy—that distinguish between two or more populations. The combination of outcomes from each force enhancing or reducing differentiation, eventually determines the pace at which populations diverge. Since decades ago, genetic markers have raised as one of the most effective tools to evaluate differentiation at the population and species levels (Avise 2004). This special issue showed the power of different traditional genetic markers, such as AFLPs (Amplified Fragment Length Polymorphisms; Medrano *et al.* 2020) and nuclear microsatellites (Muñoz-Pajares *et al.* 2020), as well as next-generation technologies, such as RAD sequencing (Lin *et al.* 2020; Silva *et al.* 2020; Palacio-Mejía *et al.* 2021), to explore molecular differentiation in plant populations.

Research articles covered the entire spectrum at which molecular differentiation currently operates or did in the past, ranging from sub-population demes, populations, within-species genetic lineages to species. In each study, researchers identified the putative forces accounting for the pervasiveness of genetic differentiation. For example, Lin *et al.* (2020) stressed that isolation by environment explained the geographical distribution of the major genetic clusters found in the wild tomato *S. pimpinellifolium*. Silva *et al.* (2020) quantified the number of putative adaptive loci conferring a stark adaptive genetic structure in the endemic tropical herb *B. carajensis* across a relatively narrow environmental gradient. Muñoz-Pajares *et al.* (2020) revealed how the extent of temporal migration due to reproductive asynchrony acted as a valve regulating the intensity of genetic differentiation within populations of the biennial plant *E. mediohispanicum*. Medrano *et al.* (2020) showed that epigenetic diversity was greater than genetic diversity in all phylogenetically closely related but ecologically disparate species. Finally, Palacio-Mejías *et al.* (2021) disentangled the combination of historical, demographic and adaptive factors accounting for the genetic divergence between two *P. hallii* varieties.

Despite the power of molecular means to disentangle genetic differentiation patterns, quantitative trait approaches are still complementary to molecular ones because they help understand mechanisms accounting for phenotypic differentiation. In fact, experimental approaches have always been a central element in evolutionary biology, even before the first use of the term ecological genetics, as shown by Turesson’s seminal work on common garden experiments (Turesson 1922) and Clausen *et al.*’s reciprocal transplant experiments (Clausen *et al.* 1940). In this special issue, several articles thoroughly considered quantitative trait differentiation across various ecological settings and highlighted the importance of quantitative trait differentiation to address fundamental ecological and evolutionary questions. Overall, these studies tackled population differentiation in a wide array of quantitative traits dealing with morphology, physiology, resource allocation, phenology and/or fitness (Matesanz *et al.* 2020; Solé-Medina *et al.* 2020; Yoko *et al.* 2020). In particular, Matesanz *et al.* (2020) stressed the important role of joint genetic differentiation and phenotypic plasticity as sources of adaptive variation in multiple traits of the annual legume *L. angustifolius*. Solé-Medina *et al.* (2020) showed how dry environmental conditions strongly determined high levels of variation in early fitness traits among *B. pendula* populations. Finally, Yoko *et al.* (2020) identified stomatal and resource allocation traits to be more important than morphological traits to understand differentiation patterns in the perennial *G. triflorum*. It is worth emphasizing the review by Honjo and Kudoh (2019) stressing the role of transcriptome data to elucidate the genetic basis of adaptive traits, which represents a promising future research line to comprehend how differentiation works at the gene level.

## A Way Forward

In spite of the technological progress in several fields (e.g., genomics, transcriptomics, phenomics, unmanned vehicles, satellite remote sensing), understanding evolutionary processes still requires field studies of natural populations. In fact, differentiation is an all-encompassing process that eventually molds organisms’ phenotypic integration and their genetic make-up. Here, we gathered a collection of papers illustrating the relevance of traditional evolutionary studies focused on plant populations, but also highlighting the integration of classical biological disciplines and state-of-the-art molecular techniques into a unique toolkit for evolutionary plant research.

Based on the papers of this special issue, we envisage two promising future research lines to keep making progress in the discipline. First, it is time to bring together ecological genetics/genomics studies combining among- and within-population variation in genetic diversity and quantitative traits at large geographical scales (see Castilla *et al.* 2020), which can be key for understanding the species’ potential response to rapidly changing selective pressures due to global climate change. These studies will require collaborative efforts among several research laboratories, similar to consortia created to generate detailed whole-genome sequence variation for several hundreds of samples across the entire species’ distribution range (as in *A. thaliana*; The 1001 Genomes Consortium 2016). Second, we also need to delve into gene and genome function in an ecological context to better comprehend the genetic mechanisms underlying responses of organisms to their natural environment (Ungerer *et al.* 2008) and the genetic basis of adaptive differentiation (Ågren *et al.* 2013). The technical advances in genomics and bioinformatics and the experience and skills achieved from model systems already enable that in several non-model plant species.

We conclude by stressing the current serious challenges menacing the existence of plant biodiversity due to the unprecedented transformation of the planet at the beginning of the Anthropocene. The continuous differentiation process affecting the evolution of plants is probably experiencing dramatic alterations with unpredictable consequences. We need population differentiation studies, like the ones in this special issue, more than ever to comprehend the causes and consequences of rapid environmental changes on plant populations to foresee—and perhaps mitigate—their effects on plant biodiversity’s fate.

## Data Availability

No data required.

## Notes

**Populations & Communities. Chief Editor:** Jean Burns
